# Identification of transcriptional regulatory nodes in soybean defense networks using transient co-transactivation assays

**DOI:** 10.3389/fpls.2015.00915

**Published:** 2015-10-27

**Authors:** Yongli Wang, Hui Wang, Yujie Ma, Haiping Du, Qing Yang, Deyue Yu

**Affiliations:** ^1^National Center for Soybean Improvement, National Key Laboratory of Crop Genetics and Germplasm Enhancement, Nanjing Agricultural UniversityNanjing, China; ^2^Biofuels Institute, School of the Environment, Jiangsu UniversityZhenjiang, China; ^3^College of Life Sciences, Nanjing Agricultural UniversityNanjing, China

**Keywords:** transcription factors, promoters, defense network, gene regulation, protoplasts

## Abstract

Plant responses to major environmental stressors, such as insect feeding, not only occur via the functions of defense genes but also involve a series of regulatory factors. Our previous transcriptome studies proposed that, in addition to two defense-related genes, *GmVSP*β and *GmN:IFR*, a high proportion of transcription factors (TFs) participate in the incompatible soybean-common cutworm interaction networks. However, the regulatory mechanisms and effects of these TFs on those induced defense-related genes remain unknown. In the present work, we isolated and identified 12 genes encoding MYB, WRKY, NAC, bZIP, and DREB TFs from a common cutworm-induced cDNA library of a resistant soybean line. Sequence analysis of the promoters of three co-expressed genes, including *GmVSP*α, *GmVSP*β, and *GmN:IFR*, revealed the enrichment of various TF-binding sites for defense and stress responses. To further identify the regulatory nodes composed of these TFs and defense gene promoters, we performed extensive transient co-transactivation assays to directly test the transcriptional activity of the 12 TFs binding at different levels to the three co-expressed gene promoters. The results showed that all 12 TFs were able to transactivate the *GmVSP*β and *GmN:IFR* promoters. *GmbZIP110* and *GmMYB75* functioned as distinct regulators of *GmVSP*α/β and *GmN:IFR* expression, respectively, while *GmWRKY39* acted as a common central regulator of *GmVSP*α/β and *GmN:IFR* expression. These corresponding TFs play crucial roles in coordinated plant defense regulation, which provides valuable information for understanding the molecular mechanisms involved in insect-induced transcriptional regulation in soybean. More importantly, the identified TFs and suitable promoters can be used to engineer insect-resistant plants in molecular breeding studies.

## Introduction

Plants have evolved a variety of active defense mechanisms to protect themselves from unfavorable environmental conditions, including pathogen infection and herbivore attack (Wu and Baldwin, 2010; Walters, 2011). A common feature of plant defense responses is that they can be divided into pre-existing constitutive defenses and induced defenses that are induced only upon attack (Agrawal, 1998; Karban and Baldwin, 2007). The induced plant defenses are mediated by a highly interconnected signaling network mainly involving jasmonate (JA), salicylate (SA) and ethylene signaling, which are likely orchestrated by important metabolic proteins (functional proteins) via the transcriptional activation of a complex regulatory network in the cell nucleus (Yang et al., 1997). In fact, such regulatory networks are critical for host defense against extracellular pathogens because they rapidly alter the expression of relevant genes. Many of these defense genes appear to be induced at the transcriptional level through the specific recognition of *cis*-acting elements in their promoters by trans-acting sequence-specific DNA-binding transcription factors (TFs; Kawaoka et al., 1994; Rushton et al., 1996; Cai et al., 2008). Thus, the transcriptional regulation of plant defense-related genes is a vital component of plant defense responses, and the elucidation of the underlying mechanisms should provide important insights into the molecular basis of induced defenses in plants.

To elucidate defense-related gene regulatory networks, components of the transcriptional regulatory systems should be identified, including genes encoding TFs and genes encoding downstream effector products. The interactions between these regulators and the genes being regulated are studied intensively, with a particular emphasis on the roles of TFs and stress-inducible promoters (Bhatnagar-Mathur et al., 2007; Liu et al., 2010). In recent years, many TFs belonging to the NAC, ERF, MYB, WRKY, and bZIP families have been identified and shown to play critical roles as either activators or suppressors in regulating the expression of defense-related genes (Eulgem and Somssich, 2007; Golldack et al., 2011). In *Arabidopsis*, at least 1510 TFs have been identified as members of different families, and many of them have shown to have multiple functions in defense responses (Qu and Zhu, 2006). Among these TFs, many members of the WRKY superfamily are involved in a multitude of plant physiological processes of defense responses to pathogens. For instance, *Arabidopsis AtWRKY70* functions an essential element of R gene-mediated resistance against the oomycete *Hyaloperonospora parasitica* by modulating cross-talk between the SA and JA signaling pathways (Knoth et al., 2007). Whereas the body of knowledge on the transcriptional regulation of plant defenses against pathogen infection is already quite large, TFs controlling plant defense responses to herbivore attack are just beginning to be identified. The best-studied TFs involved in plant–insect interactions are MYC2 and several WRKYs (Dombrecht et al., 2007; Skibbe et al., 2008). *AtMYC2*, an *Arabidopsis thaliana* TF, was reported to function downstream of JA and to regulate JA-mediated herbivore resistance in plants (Dombrecht et al., 2007). Because the soybean genome is publicly available, a massive number of soybean genes have been comprehensively annotated as putative TFs. However, relatively few TFs that regulate plant-induced defenses have been identified to date (De Geyter et al., 2012).

The characterization of TFs involved in the regulation of plant genes has led to extensive promoter analyses to search for *cis*-regulatory elements in plant defense regulatory systems (Higo et al., 1999; Chakravarthy et al., 2003). The coexistence of multiple *cis*-regulatory elements in specific gene promoters and the known functions of these elements in response to environmental stimuli also suggest the complexity of gene regulatory networks. Two groups of pathogen-inducible *cis*-acting elements, GCC-like elements (Ohme-Tagaki et al., 2000) and W boxes (Robatzek and Somssich, 2002), have been well studied. W-box sequences are specifically recognized by WRKY proteins and are necessary for the inducible expression of many defense-related genes, including several pathogenesis-related (PR) genes and the regulatory *NPR1* gene, that contain W-box elements in their promoters (Rushton et al., 1996; Yu et al., 2001). A similar element has been reported to direct JA and elicitor-responsive expression (JERE; AGACCGCC) (Menke et al., 1999), and another element (DRE; TACCGAC) directs cold-, salt stress-, and dehydration-responsive expression (Yamaguchi-Shinozaki and Shinozaki, 1994). Recently, a similar GCC-like element, called box S (AGCCACC), was identified that directs expression by fungal elicitors (Kirsch et al., 2000). All of the above studies have contributed greatly to the development of various binding site databases, such as PLACE (a database for plant *cis*-acting regulatory elements) and PlantCARE (a database for the analysis of putative binding sites in promoters) (Higo et al., 1999; Lescot et al., 2002).

We previously initiated a program to study the induced defense responses of soybean against the common cutworm (*Spodoptera litura* Fabricius, CCW). Differential patterns of induced resistance to CCW were identified in both resistant and susceptible lines, and an integrated view of the transcriptional changes associated with the induced defense responses was obtained from a comparative transcriptome analysis using microarrays (Wang et al., 2014). The genes significantly induced or repressed by CCW attack were involved in several different functional categories, primarily signaling related to defense and/or stress, transcriptional regulation and secondary metabolism (Wang et al., 2014). Recently, two JA-responsive genes (a soybean vegetative storage protein [VSP]-encoding gene [*GmVSP*β] and an NADPH:isoflavone reductase-encoding gene [*GmN:IFR*]) were further functionally analyzed in transgenic tobacco and were shown to be associated with CCW resistance (Wang et al., 2015). However, it remains unclear how these genes are transcriptionally activated in response to insect treatment in soybean and whether these genes function in a coordinated manner with the complex complement of other compartments in the plant cell.

The similar expression patterns of TFs and their downstream target genes suggest that a complicated gene regulatory network is involved in CCW-induced pathways in resistant soybean lines, although the details of this molecular process are not yet fully understood. Thus, we chose to address the question of which specific TFs deduced from microarray data are key regulatory factors of soybean-induced defenses. The promoters of the well-characterized *GmVSP*α*/*β and *GmN:IFR* genes were analyzed using the PLACE and PlantCARE promoter databases. 12 TF genes including four WRKYs, three MYBs, three NACs, one bZIP and one DREB were isolated and studied in the *Arabidopsis* transient assay system to examine the different effects of these TFs on the three promoters mentioned above. The results reveal that new regulatory players in soybean defenses against generalist herbivores, and it highlights the predominant role of the inducible *GmVSP*β and *GmN:IFR* promoters. These findings may be valuable for regulating the expression of resistance genes against insect and/or pathogen attacks.

## Materials and Methods

### Expression Cluster Analysis

Forty-five TF genes were screened from a previously reported analysis of two soybean lines’ transcriptome responses to the CCW (Wang et al., 2014). Two well-characterized functional genes that were specifically induced after CCW feeding in a resistant soybean line and a third, related functional gene that was induced in susceptible soybean lines were also involved in this study. The microarray data with accession number GSE51823 was obtained from the National Center for Biotechnology Information (NCBI), Gene Expression Omnibus (GEO^1^). The gene codes of all the TFs studied, referred to as Gma numbers, were retrieved from a soybean genome sequence database^2^ (Schmutz et al., 2010). To compare expression profiles, the expression values of all 45 TFs and the three functional genes were normalized and transformed into logarithmic (base 2) values. Subsequently, an average linkage hierarchical cluster using an uncentered correlation metric was applied using Cluster v3.0 (Eisen et al., 1998), and the results were visualized using Java TreeView v1.0.5 software (Saldanha, 2004).

### cDNA Cloning of Transcription Factor Candidates and Sequence Analysis

We cloned the full-length cDNAs of 12 candidate TFs from cDNA libraries prepared from the leaves of a resistant soybean line (WX) fed on by CCW for 48 h (Wang et al., 2015). Candidate TFs were selected on the basis of their putative functions and expression patterns. The primer pairs for each TF were designed from their reference sequences in the soybean genome sequence database and the NCBI mRNA database; these accession numbers are listed in **Table 1** (the primers are listed in Supplementary Table S1). All PCR products were purified, cloned into the pMD19-T Easy vector (Takara, Dalian, China), and sequenced (Invitrogen, Shanghai, China). The sequence information was used to isolate full-length cDNAs for the corresponding TFs. The sequences of all TFs were consistent with their reference sequences in the soybean genome sequence database. The deduced amino acid sequence of each cloned TF was used to search the corresponding conserved domain in the NCBI Conserved Domain Database^3^. Nuclear localization signals were predicted using PSORT^4^. Protfun^5^ was used to predict the putative function of each TF. Additionally, the amino acid sequences of the identified TFs were subjected to Basic Local Alignment Search Tool (BLASTX) similarity searches against the GenBank database to search for putative orthologs of the TFs. Identity, similarity and gap percentages between our TFs and their orthologs were calculated using the FASTA program (Pearson, 1990). To explore the evolutionary relationships between the soybean and other plant TFs, we constructed a neighbor-joining (NJ) tree from the amino acid sequences of the aligned soybean and other plant TFs using the ClustalX2 program (Larkin et al., 2007) and MEGA version 4.1 (Kumar et al., 2008). The bootstrap trials were replicated 1000 times to derive confidence values for the phylogeny analysis.

**Table 1:** Expression level and bioinformatic analysis of 12 soybean transcription factors.

Item TFs	Fold change^a^		CDS(ID)	Amino Acids	Sub-location	Function prediction	Domain	Homologous genes
	MA-R^b^	MA-S^c^						
*GmWRKY28*	3.58	None	NM_001250598.1	293	Nuclear	Replication_and_transcription	WRKY superfamily (140–197)	At WRKY57
*GmWRKY21*	4.90	None	NM_001250398.1	197	Nuclear	Transcription_regulation	WRKY superfamily (110–169)	At WRKY51
*GmWRKY39*	3.48	1.24	XM_003519225.2	580	Nuclear	Transcription_regulation	WRKY superfamily1 (236–294; 408–466)	At WRKY33;
*GmWRKY20*	6.56	0.59	XM_003530331.1	359	Nuclear	Replication_and_transcription	WRKY superfamily (127–189)	At WRKY53
*GmbZIP110*	2.00	0.94	NM_001250218.2	168	Nuclear	Transcription_regulation	B_zip1 superfamily (30–95)	OCSBF-1; AtZIP1
*GmDREB1*	2.13	1.51	NP_001235779.1	174	Nuclear	Replication_and_transcription	AP2 superfamily (45–103)	At RAP2-1
*GmNAC34*	5.25	1.03	NM_001251326.2	302	Nuclear	Enzyme	NAM superfamily (11–135)	AtNAC22
*GmNAC3*	3.16	0.77	AAY46123.1	317	Nuclear	Energy_metabolism	NAM superfamily (14–140)	AtNAC72; AtNAC19
*GmNAC26*	11.10	None	NM_001251275.1	279	Nuclear	Transcription_regulation	NAM superfamily (9–86)	AtNAC29; AtNAC25
*GmMYB50*	2.04	0.70	NM_001251158.1	297	Nuclear	Regulatory_functions	SANT superfamily (7–49; 59–102)	AtMYB44
*GmMYB73*	2.25	None	NM_001250849.1	74	Plasma membrane	Cell_envelope	SANT superfamily (27–66)	AtTRY; AtCPC
*GmMYB75*	2.12	None	NM_001248819.1	306	Nuclear	Central_intermediary_metabolism	SANT superfamily (26–72; 126–170)	AmDIV

*^a^Fold change based on microarray data (GSE51823); ^b^Fold change values of the induced resistant soybean line compared with non-induced sample; ^c^Fold change values of the induced susceptible soybean line compared with non-induced sample.*

### Promoter Isolation and Analysis

The *GmVSP*α, *GmVSP*β, and *GmN:IFR* promoters were amplified from WX genomic DNA using specific primers. The primer design was based on 2-kb reference sequences upstream of the inferred translational start sites in the soybean genome sequence database (Supplementary Table S2). Genomic DNA was extracted from young leaves of the resistant soybean line WX using a DNA plant extraction kit (Tiangen). A PTC-225 thermal cycler (MJ Research, Watertown, MA, USA) was used to perform PCR in a 50-μl reaction volume with KOD polymerase (Toyobo, Japan), following the manufacturer’s recommendations. The cycling program consisted of one cycle at 94°C for 2 min; 35 cycles of 94°C for 15 s and 68°C for 2 min; and one cycle of 4°C for 10 min. The amplification products (1450 bp for *GmVSP*α, 1981 bp for *GmVSP*β and 1812 bp for *GmN:IFR*) were separated by electrophoresis on a 1% agarose gel, and bands of the expected size were excised and purified using the AP-GX-4 PCR gel purification kit (Axygen, China). Three sample PCR products were purified and sequenced at Invitrogen (Shanghai, China). Putative cis-acting regulatory elements were identified through searches against both the PlantCARE and PLACE databases (Higo et al., 1999; Lescot et al., 2002).

### Design of TF Effector and LUC Reporter Constructs

Effector constructs and LUC reporter constructs were constructed from the pCaMV35S-*LUC* basal expression plasmid, which contains the 35S promoter of cauliflower mosaic virus, multiple cloning sites, a LUC reporter gene and NOS (Supplementary Figure S1). This plasmid was generated from pRD29A-*LUC* (EF090409) by replacing the RD29A promoter with an 835-bp CaMV35S promoter cloned from the pBI221 (AF502128.1) plasmid. To generate the effector constructs, the coding regions of each of the 12 TFs we identified were amplified by PCR using a new pair of specific primers containing an additional 20 complementary oligonucleotides overlapping 5′ flanking NcoI and 3′ flanking EcoRI restriction sites. Each TF coding region was then inserted into the pCaMV35S-*LUC* vector to replace the ORF of the LUC gene using the ClonExpress II One Step Cloning Kit (Vazyme Co. Ltd., China) following standard procedures. Similarly, the reporter constructs were generated by PCR-amplifying the ∼1.5-kb identified promoter of *GmVSP*α, *GmVSP*β or *GmN*:*IFR* using a new pair of specific primers containing an additional 20 complementary oligonucleotides overlapping the 5′ flanking PstI and 3′ flanking NcoI restriction sites. Each amplicon was then inserted into the pCaMV35S-*LUC* vector to replace the 35S promoter, again using the ClonExpress II One Step Cloning Kit. The fidelity of all the constructs was confirmed by restriction and sequence analyses. This design allowed the 12 TF genes to be constitutively expressed from the effector constructs under the control of the 35S promoter. In contrast, the *GmVSP*α, *GmVSP*β, or *GmN:IFR* promoter drove the expression of the LUC reporter gene from the respective reporter construct alone or under specific conditions.

### Protoplast Isolation

Protoplasts isolated from *Arabidopsis* leaves are useful materials in plant research. One application, the transient expression of recombinant genes using *Arabidopsis* mesophyll protoplasts, is commonly used to study promoter activity and *in vivo* protein–protein interactions (Abel and Theologis, 1994). The *Arabidopsis* genotypes used in this study were WT of the ecotype Col-0. *Arabidopsis thaliana* plants were grown in a mixture of vermiculite, perlite, and peat moss at a 1:1:1 ratio in an environmentally controlled chamber with a long photoperiod (16 h light and 8 h dark) at 22°C. Four-week-old healthy *Arabidopsis* optimal true leaves (fifth, sixth, and seventh) were selected for protoplast isolation. Protoplasts were isolated from the *Arabidopsis* leaves according to a procedure described by Sheen (2002) using a protoplast enzyme solution containing 1% cellulase, 0.1% pectolyase Y-23, 0.4 M mannitol, 20 mM KCl, 20 mM MES pH 5.7, 10 mM CaCl_2_, 5 mM 2-mercaptoethanol and 0.1% BSA. The protoplasts were shown to be intact and viable under fluorescence microscopy (400x; Supplementary Figure S2).

### Protoplast Transient Transformation and LUC Assays

A variety of different transfection techniques, such as PEG-mediated transformation, electroporation, and microinjection, can be used to introduce DNA into plant protoplasts. The most commonly used technique, PEG-mediated transformation, is simple and efficient, allows the simultaneous processing of many samples, and yields a transformed cell population with high survival and division rates (Mathur and Koncz, 1998). Thus, the transformation of isolated *Arabidopsis* protoplasts was performed using the PEG-mediated method, as described by Yoo et al. (2007). In the co-transfection assay, 2 × 10^5^/ml protoplasts in 120 μl were transfected with 15 μl (∼10 μg) of effector plasmid containing each of the 12 TF genes gently mixed with 15 μl (∼10 μg) of reporter plasmid containing the *GmVSP*α, *GmVSP*β, or *GmN*:*IFR* promoter. Then, 220 μl of 40% PEG6000 (Sigma Aldrich) was added, followed by a 30-min incubation at room temperature. The transformed protoplasts were pelleted by centrifugation at 100 × *g* for 5 min at 4°C, resuspended in 800 μl of W5 solution (154 mM NaCl, 125 mM CaCl_2_, 5 mM KCl, and 2 mM MES) containing 0.4 M mannitol, and incubated for 48 h at room temperature in the dark. The protoplasts were harvested by centrifugation at 100 × *g* for 5 min at room temperature. The pellet was washed twice with 2 ml of W5 solution and resuspended in Luciferase Cell Culture Lysis Reagent (CCLR) from the Luciferase Assay System (Promega). LUC activity was measured using a luminescence reader (TD-20/20; Promega) according to the manufacturer’s guidelines. In each experiment, the expression level of the LUC reporter gene in protoplasts transfected with a reporter plasmid containing the GmVSP*α*, GmVSP*β* or GmN:IFR promoter alone (without the effector) was used as the control value. All transfection experiments were performed at least three times. The experimental data were statistically analyzed with two-tailed correlation tests among groups using SPSS13.0 (SPSS). Statistical significance was set at *p* < 0.05, with *p* < 0.01 indicating highly significant results.

## Results and Discussion

### Co-regulated Transcription Factors from the Herbivory-Induced Soybean Transcriptome

**FIGURE 1:**
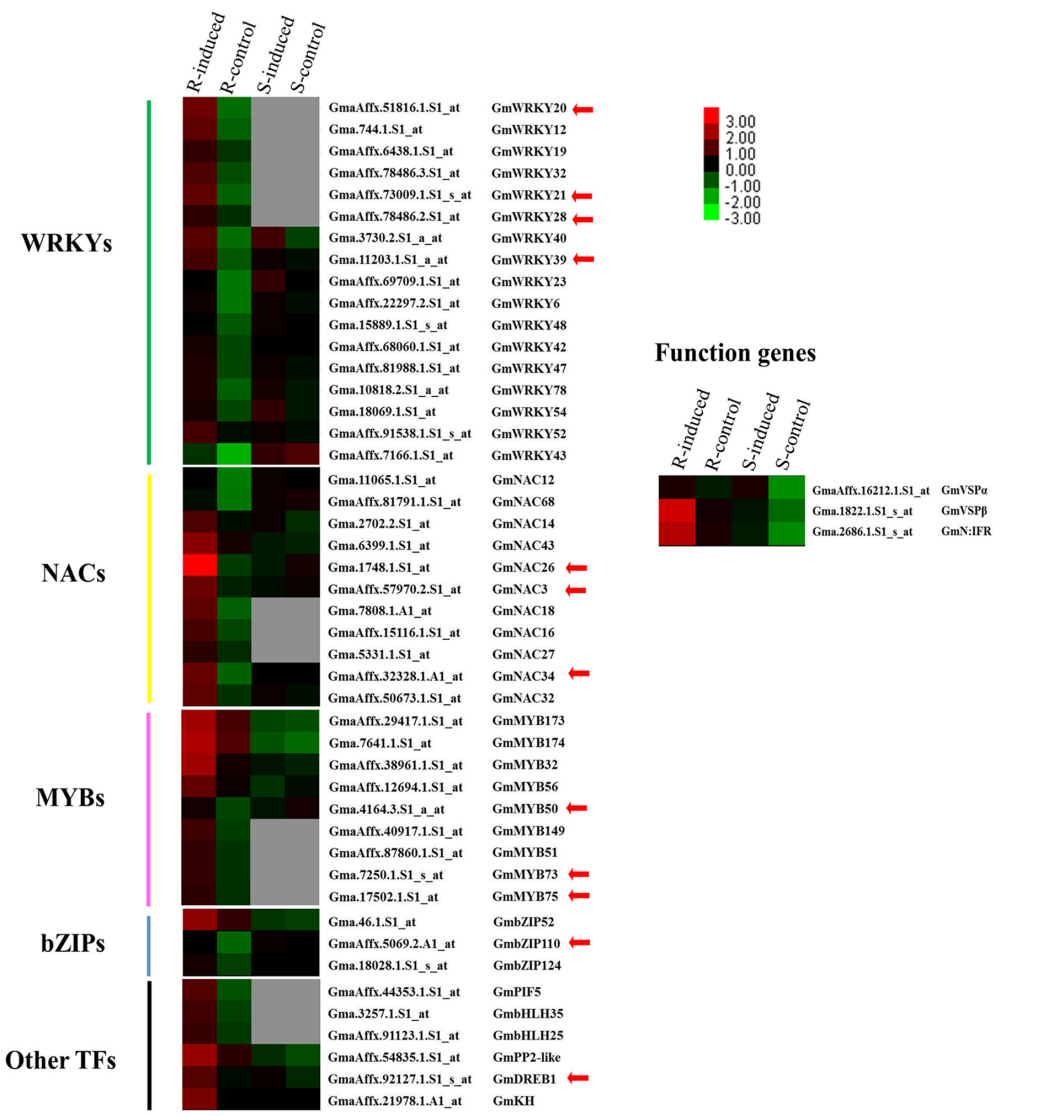
**Expression pattern of 45 TFs and three defense related function genes in the resistant (R) and susceptible lines (S) with or without induction by common cutworms.** The heat map visualizes the expression profiles of these differentially expressed genes from microarray data (GSE51823, Wang et al., 2014). Red and green indicate high and low expression levels and no detectable expression is in gray.

Plants experience various environmental stresses, including pathogen infection and herbivore attack. During the response and adaptation to herbivory stresses, many genes related to specific biochemical and physiological processes are activated at the transcript level in many plant species (Arimura et al., 2000; Hui et al., 2003; Venu et al., 2010). Our previous microarray-based transcriptome analyses of two CCW-induced soybean lines provided a wealth of global gene expression data and identified many stress/defense-related genes that are commonly or uniquely induced at the peak time of induced resistance in resistant and susceptible soybean lines. The resistant line gives a longer, higher level of induced resistance to CCW, whereas the susceptible line gives a shorter, weaker level of induced resistance to the same insects (Wang et al., 2014). Therefore, it was not surprising that some commonly regulated functional genes, represented by *GmVSP*β and *GmN:IFR*, were activated to a greater degree in the resistant soybean line than in the susceptible line (**Figure 1**). Whereas the body of knowledge on the functional roles of these stress/defense-related genes is already quite large, the regulatory networks involved in soybean insect defense systems and the molecular links between regulatory factors and functional genes are just beginning to be revealed. In the same microarray data, a group of genes encoding known or putative TFs was strikingly observed to be uniquely activated in the resistant line at the same time as *GmVSP*β and *GmN:IFR* (**Figure 1**). The co-regulated expression patterns of the TFs and the two functional genes suggested that both groups of genes may be involved in the same network modules and thus are likely related to CCW-induced resistance in the resistant soybean line. The 45 co-regulated TFs belong to many different families, including 17 WRKYs, 11 NACs, 9 MYBs, 3 bZIPs and several others (**Figure 1**). This large number of TF genes may reflect the complexity of plant defense regulation, and various TFs may have different functions in this process. The expression levels of these TFs all showed a FC greater than 2.0 (*p* < 0.05) in the soybean line with induced resistance, whereas these values did not change or were missing in the susceptible line. This finding suggests that the resistant plants triggered massive and stronger transcriptional reprogramming to produce longer and higher levels of induced resistance against CCW; this response is similar to plant defense responses against aphids and microbial pathogens (Gao et al., 2010; Madrid et al., 2010).

Quantitative differences in the expression of TF genes can result in dramatic differences in DNA binding and regulatory capability. In *Arabidopsis*, it was found that a large proportion of genes with different expression patterns among different accessions encode TFs (Schmid et al., 2005). Numerous TFs have been shown or suggested to be key regulators of the plant defense response, and most are involved in defense responses to abiotic stress and pathogens (Hammond-Kosack and Parker, 2003; Eulgem and Somssich, 2007; Oñate-Sánchez et al., 2007). For instance, members of the WRKY family of TFs are notable for responding to pathogens, and members of the NAC, bZIP and MYB families have well-characterized roles in abiotic stress responses (Eulgem and Somssich, 2007; Yánez et al., 2009; Rahaie et al., 2010; Wang et al., 2013). Unfortunately, little is known regarding the regulatory roles of the 45 TFs that we identified as related to insect resistance in soybean, except for several genes that have been shown to regulate the response to other stresses. Among the latter group of genes, *WRKY20, WRKY21*, and *DREB1* have been previously reported to regulate tolerance to stress in transgenic plants under drought, salt or cold conditions (Zhou et al., 2008; Luo et al., 2013; Kidokoro et al., 2015). Another member, *WRKY39*, has been implicated in induced resistance to *Sclerotinia sclerotiorum* in oxalate oxidase transgenic soybean (Calla et al., 2014). Members of other families, such as *NAC3*, are strongly induced by osmotic stress, ABA, JA or salinity (Pinheiro et al., 2009), and *MYB73* was found to function in lipid accumulation (Liu et al., 2014b). These six TFs with known functions, along with the remaining 39 TFs that have not been experimentally analyzed, may play important roles in controlling defense gene expression and CCW-induced resistance responses in soybean. Some of these genes, together with their targets, may constitute key regulatory switches in multiple molecular mechanisms of insect defense. Therefore, understanding the relationships among these regulatory switches is important for dissecting and properly reconstructing the gene regulatory networks.

### Identification of 12 Transcription Factors as Candidate Regulators of the Defense Response

**FIGURE 2:**
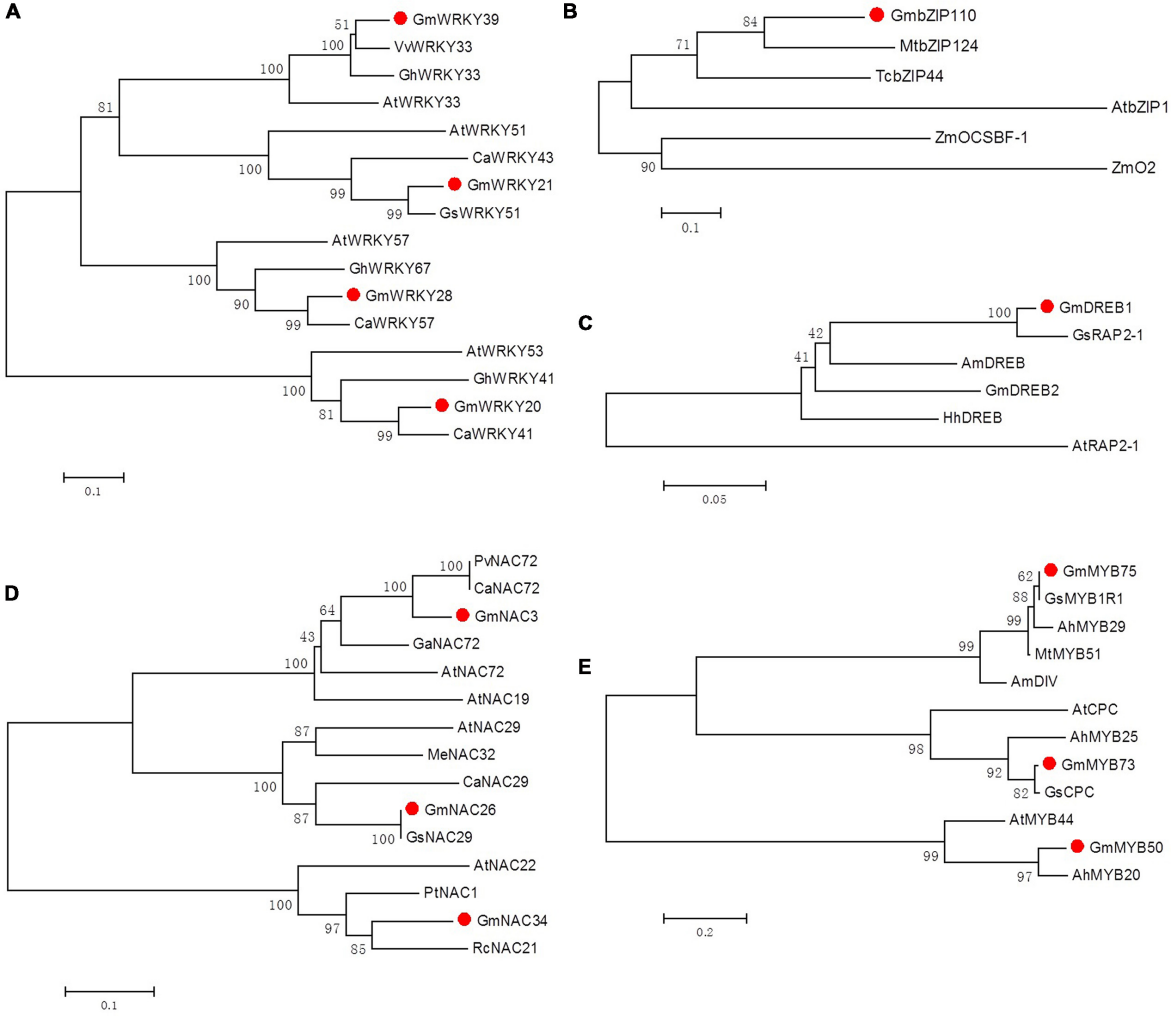
**Phylogenetic tree analyses of soybean WRKY **(A)**, bZIP **(B)**, DREB **(C)**, NAC **(D)** and MYB **(E)** transcription factors and their homologous genes.** *Gm, Glycine max*; *Gs, Glycine soja*; *At, Arabidopsis thaliana*; *Zm, Zea mays*; *Am, Antirrhinum majus*; *Pv, Phaseolus vulgaris*; *Ca, Cicer arietinum*; *Ah, Arachis hypogaea*; *Mt, Medicago truncatula*; *Ga, Gossypium arboretum*; *Gh, Gossypium hirsutum*; *Me, Manihot esculenta*; *Rc, Ricinus communis*; *Hh, Halimodendron halodendron*; *Pt, Populus trichocarpa*; *Tc, Theobroma cacao*; *Vv, Vitis vinifera*. Accession numbers of 12 soybean transcription factors are listed in **Table 1** and of the homologous genes are listed in Supplementary Table S3.

TFs commonly bind to highly conserved sequence motifs in the promoters of defense-related genes, suggesting the direct involvement of TFs in the regulation of the plant defense response (Rushton et al., 1996; Eulgem et al., 1999). Because plant adaptability to various stresses is mainly controlled by the regulation of TFs, substantial research has focused on the regulatory roles of plant TFs under different stress conditions (Rushton and Somssich, 1998). However, the precise functions of these TFs have not been adequately identified. It is well known that a gene’s expression pattern can offer important clues regarding its function. Here, we hypothesized that the expression of TF genes as potential regulators would precede the expression of their targets. Using transcriptome profiling, we identified 45 TFs that were induced in response to herbivory by CCW. Five of these genes, *WRKY20, WRKY21, WRKY39, DREB1* and *NAC3*, were demonstrated or suggested to play significant roles in response to abiotic or disease stresses. These stresses, especially drought and disease, often coincide and interact with herbivory stress (Taylor et al., 2004; Xiao et al., 2013). The other gene *MYB73* was found to have functions in regulation of lipid metabolism, which are not only useful for energy complement but also involved in regulating cell signaling process (Liu et al., 2014b). Thus, we focused our efforts on the further analysis of these six genes and six other TFs with unknown roles and functions; overall, these genes included four WRKYs, three MYBs, three NACs, one bZIP and one DREB (**Figure 2**).

**FIGURE 3:**
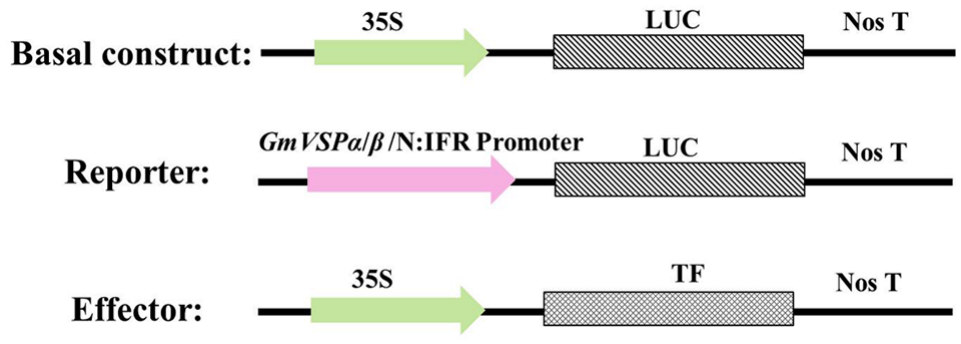
**Schematic representation of basal construct, reporter and effector constructs used in the transient transactivation assays.** Plasmid pCaMV35S-*LUC* was used as basal construct, each of the GmVSP*α*_pro_:*LUC*, GmVSPβ_pro_:*LUC* or GmN:IFR_pro_:*LUC* fusion was used as the reporter construct, and each of the 35S_pro_:TF (WRKY28, 21, 39, or 20; NAC 34, 3 or 26; MYB 50, 73 or 75; bZIP110 or DREB1) was used as effector construct.

Twelve full-length cDNA sequences encoding these TF proteins were isolated from cDNA libraries derived from CCW-attacked leaves of the resistant line (Wang et al., 2014). Twelve effector constructs were generated for subsequent analyses by replacing the LUC cDNA fragment of the basal construct pCaMV35S-LUC with these cDNA fragments (**Figure 3**). The identified cDNAs encode proteins of 74–580 amino acid residues in length. Further sequence analysis identified one or two specific domains in each TF protein sequence, which indicated the different groups to which these TFs belong. For example, most of the selected WRKYs (*GmWRKY28, GmWRKY21*, and *GmWRKY20*) contain one WRKY domain and belong to WRKY group II or group III, except for *GmWRKY39*, which contains two WRKY domains and belongs to WRKY group I (**Table 1**). Because TFs mainly exert their activity in the nucleus, the subcellular location of each TF protein was identified using PSORT. The results revealed that most of these candidate TFs were located in the nucleus; the only exception, *GmMYB73*, was located in the plasma membrane, which might be explained by the small size of the GmMYB73 protein. In addition, it was reported that GmMYB73 functioned through the interaction with other TFs that has a nuclear localization signal (Liu et al., 2014b). Likewise, the cellular roles of most of these TF proteins were predicted to be related to transcriptional regulation, replication and transcription (**Table 1**).

After the 12 TFs were translated, homology searches (BLASTX) of their ORFs against the NCBI non-redundant database returned sequences of putative TFs from various plant species, such as *Cicer arietinum, Medicago truncatula, Arabidopsis thaliana, Gossypium hirsutum*, and *Vitis vinifera*. To gain insights into the biological functions of these genes, five phylogenetic trees of different TF families were constructed and visualized in **Figure 2**. The generated trees revealed that each TF was clustered with its homologs on a separate branch, rather than with members of the same soybean family, which means that there is no redundancy between the selected 12 TFs. Most of the 12 TFs were clustered closest with homologous TFs from leguminous plants, such as *GmWRKY20* with *CaWRKY41, GmbZIP110* with *MtbZIP124, GmDREB1* with *GsRAP2-1, GmMYB75* with *MtMYB51, GmMYB50* with *AhMYB20*. The only two exceptions were GmNAC34 and GmWRKY39, the latter one formed a closest cluster with VvWRKY33, which has recently been identified to be involved in the regulation of grapevine (Vitis vinifera) defense against the oomycete pathogen *Plasmopara viticola* (Merz et al., 2015). Although a large part of of those most homologous TFs were putative new TFs or TFs with unknown functions, some *Arabidopsis* orthologous TF proteins have been well characterized experimentally for known functions. Most of these *Arabidopsis* homologous genes were found to be more than 50% identical to the target TF. Among them, GmWRKY20 was grouped with *Arabidopsis* AtWRKY53, which plays a regulatory role in the early events of leaf senescence and represents a convergence node between senescence and pathogen responses (Hinderhofer and Zentgraf, 2001; Miao and Zentgraf, 2010). GmNAC26 was clustered with AtNAC29, which also plays an important role in leaf senescence (Guo and Gan, 2006), thus indicating an interrelationship between plant defense regulatory responses and leaf senescence processes. Overall, the classification of the 12 TFs in the phylogenetic tree suggests that the soybean mechanism of defense against CCW is regulated by various TF proteins of divergent structure, rather than by a specific type of conserved family or TF. Our study of the 12 soybean TFs on their regulatory roles in soybean defense networks may also be useful for the functional prediction of those unknown homologous TFs.

### Isolation and Characterization of Three Herbivory-regulated Promoters

Understanding the transcriptional regulation of defense-associated genes is important for improving insect resistance mechanisms in plants. We selected 12 TF genes related to CCW resistance for further analyses and hypothesized that these TF genes may regulate the expression of functional genes involved in the molecular response to CCW feeding in soybean. These TFs share common expression patterns in response to CCW feeding with two functional genes, *GmVSP*β and *GmN:IFR*, which have been identified as playing a critical role in CCW resistance in transgenic tobacco (Wang et al., 2015). The analysis of the *GmVSP*β gene revealed an isoform, designated *GmVSP*α, that shares high sequence identity (80%) with *GmVSP*β in the coding regions but showed a different expression pattern in response to CCW feeding in two soybean lines. The transcription of *GmVSP*β was strongly induced by CCW in the resistant soybean line, while *GmVSP*α transcription was induced in the susceptible line only, which did not correlate with the expression patterns of the 12 TFs.

To learn more about the similar and different regulatory mechanisms of *GmVSP*α, *GmVSP*β and *GmN:IFR* underlying the induced defense responses, we isolated the promoters of these three genes and performed a sequence analysis to detect putative cis-elements. Functional cis-regulatory elements in plant promoters are typically found within the first 1 kb upstream of the ATG translation start site (Rombauts et al., 2003). Therefore, fragments of the *GmVSP*α, *GmVSP*β, and *GmN:IFR* promoters over 1 kb in length were successfully obtained from the genome of the resistant soybean line. A sequence comparison between the isolated fragments and sequences obtained from soybean genome database demonstrated 100% identity for *GmVSP*β and *GmN:IFR* and 98.4% identity for *GmVSP*α. An alignment of the 1300-bp promoter sequences of *GmVSP*α and *GmVSP*β showed that, although the sequences of their gene-coding regions were similar, their promoter sequences had only 47.4% identity. This result indicated that the different responses of *GmVSP*α and *GmVSP*β to CCW in two soybean lines may have arisen from their different promoter sequences and the resulting regulatory element differences.

An analysis of the *GmVSP*α, *GmVSP*β, and *GmN:IFR* promoters using the PLACE and Plant-CARE databases revealed a number of potential *cis*-regulatory elements, including basal elements, such as the TATA-box, CAAT-box and TA-rich regions, as well as elements related to defense/stress, phytohormones, and growth and development. As most defense-related genes are activated more quickly and strongly in resistant interactions, elements involved in defense/stress were over-represented along the *GmVSP*β and *GmN:IFR* promoter sequences; these elements included TC-rich repeats and silencing element-binding factor (SEBF) motifs, both of which are involved in induced disease responses (Xu et al., 2010, 2011; Germain et al., 2012). As expected, various putative transcription factor-binding sites (TFBSs) involved in pathogen and other stresses were predicted at multiple sites in the promoter regions of *GmVSP*α, *GmVSP*β, and *GmN:IFR* (**Table 2**). The analysis identified far more WRKY-binding sites, MYB-binding sites and NAC-binding sites in the *GmVSP*β and *GmN:IFR* promoters, more bZIP-binding sites in the *GmVSP*α and *GmVSP*β promoters, and numerous DREB-binding sites present only in the *GmVSP*β promoter (**Table 2**). The different numbers of TFBSs present in the promoter of each gene may define their differential promoter activity, with different combinatorial control exerted by multiple interacting TFs. In addition to the above sites, the *GmVSP*β and *GmN:IFR* promoters were found to contain multiple copies of hormone-responsive cis-regulatory elements, such as JA-induced T/G-box, SA-responsive TCA-element and cytokinin-regulated ARR1AT (**Table 2**). This finding suggested the possibility that cross-talk between different signaling pathways regulates *GmVSP*β and *GmN:IFR*. In summary, the coexistence of multiple different and some common *cis*-regulatory elements in the three genes suggested that distinct modes of transcriptional regulation might exist for the expression of *GmVSP*α, *GmVSP*β and *GmN:IFR*. The promoters of these genes are valuable additions to the study of signaling and transcriptional activation during plant–insect interactions.

**Table 2:** Comparison of the number of cis-acting elements in the promoter regions of *GmVSP*α, *GmVSP*β, and *GmN:IFR.*

*Cis*-element type	*Cis*-element name	*GmVSP*α*^a^*	*GmVSP*β	*GmN:IFR*	Putative binding TF or Function
Basal element	TATA-box	44	13	9	Transcription start core element
	TA-rich region	17	0	0	Enhancer
	CAAT-box	22	37	47	Enhancer
Defense and Stress	ABRERATCAL	0	0	1	Ca^2+^-responsive cis elements
	TC-rich repeats	1	2	2	*Cis*-acting element involved in defense and stress responses
	SEBF	0	1	1	*Cis*-acting element involved in insect and disease defense
	HSE	1	2	6	*Cis*-acting element involved in heat stress responsiveness
	LTR	1	2	0	Low-temperature responsive element
	BIHD1OS	1	1	0	Disease resistance responses
	MYCATRD22	0	1	0	Binding site of dehydration-resposive gene
	WRKY binding sites	4	17	19	W box element; Elicitor responsive element; SA-induced WRKY binding sites
	MYB binding sites	10	12	25	Regulation of flavonoid biosynthesis, water stress
	bZIP binding sites	21	20	6	Induced by ABA; SAR related Motif
	NAC binding sites	4	6	5	Early responsive to dehydration
	DREB binding sites	0	8	0	Regulated by temperature induced by ABA
Phytohormone	ARR1AT	9	17	22	Cytokinin-regulated transcription factor binding site
	ARFAT	0	1	1	ARF (auxin response factor) binding site
	T/G-box	0	0	1	Involved in jasmonate (JA) induction of proteinase inhibitor II
	ERE	0	1	1	Ethylene-responsive element
	TCA-element	1	3	1	*Cis*-acting element involved in salicylic acid responsiveness
	ABRE	1	1	0	*Cis*-acting element involved in the ABA responsiveness
	GARE-motif	1	2	0	GA-responsive element
Growth and development	A-BOX	0	0	1	Sequence conserved in alpha-amylase promoters
	Light responsive elements	28	35	17	Mainly including I-BOX
	CCGTCC-box	0	0	1	Related to meristem specific activation
	Skn-1_motif	0	1	1	Required for endosperm expression
	GCN4_motif	1	2	0	*Cis*-regulatory element involved in endosperm expression
	AACA_motif	0	0	1	Involved in endosperm-specific negative expression
Others	5UTR Py-rich stretch	0	1	0	*Cis*-acting element conferring high transcription levels
	GMHDLGMVSPB	0	1	0	Found in domain of the soybean VSPβ promoter

*^a^Copy number of predicted cis-elements in relative promoter.*

### Quantitative Analysis of Regulated Promoter Activity Associated with Induced Insect Resistance

**FIGURE 4:**
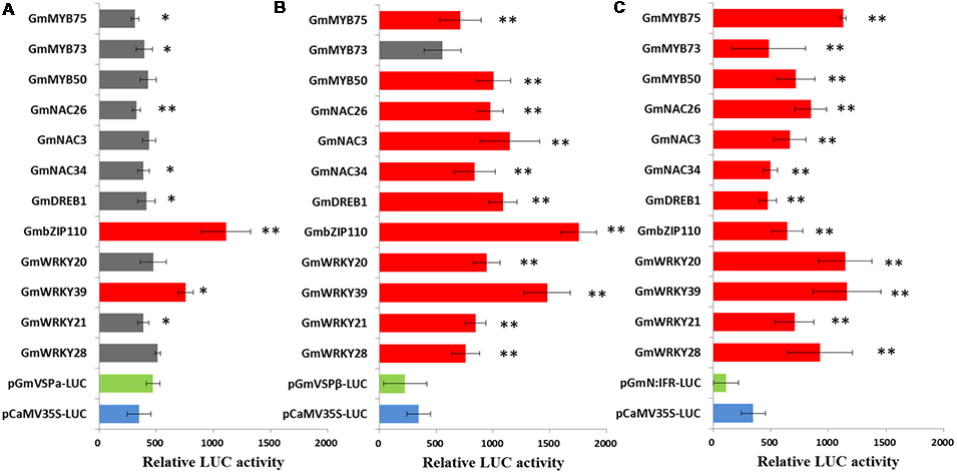
**Transient transactivation assays were performed by co-transforming reporter plasmids GmVSP**α_pro_**:*LUC***(A)**, GmVSP**β_pro_**:*LUC***(B)** and GmN:IFR_pro_:*LUC***(C)** with each of the effector plasmids at a molar ratio of 1:1 into *Arabidopsis* leaf protoplasts.** Relative LUC activity of each assay was tested and compared with the control without effector with a *t*-test. Bars indicate the standard errors of three replicates. ^∗^ Significant (*P* < 0.05), ^∗∗^ Highly Significant (*P* < 0.01). Columns of TFs with significant transactivation effects compared with the control without effector were filled with red and columns of TFs with no significant transactivation effects were filled with gray.

A previous comparative transcriptome analysis of two soybean lines in response to CCW feeding provided valuable information for understanding stress-responsive gene regulatory networks. Components of the transcriptional regulatory systems were identified, including genes encoding various TFs and genes encoding downstream effectors. To reduce the amount of data and network complexity, the regulation of the activity of the *GmVSP*α, *GmVSP*β, and *GmN:IFR* promoters by each of the screened 12 TFs was quantified and compared using a transient transactivation assay in *Arabidopsis* protoplasts, which had been previously used successfully to investigate TF interactions with plant promoters (Yi et al., 2010). Protoplasts were co-transfected with luciferase (LUC) reporter constructs containing the full-length promoter region of *GmVSP*α, *GmVSP*β, or *GmN:IFR* and effector plasmids containing the cDNA of each of the 12 screened TFs fused to the CaMV35S promoter—a well-characterized constitutive promoter that confers strong transgene expression in dicot species (Benfey and Chua, 1990). Protoplasts transfected with each reporter construct alone, including the unmodified CaMV35S::LUC construct, were used as controls (**Figure 3**). For each control and co-transfection sample, LUC activity was measured to estimate promoter activity. The results showed that all the promoters produced sufficiently strong LUC expression for detection in the transfected protoplasts; however, the *CaMV 35S, GmVSP*α, *GmVSP*β, and *GmN*:*IFR* promoters drove inconsistent levels of LUC expression (**Figure 4**). Promoters that are useful for plant transgene expression can be classified as constitutive or inducible (Weising and Kahl, 1991). Inducible promoters usually drive a weaker level of expression of a foreign gene compared with constitutive promoters (Kasuga et al., 2004). Of the four promoters, the *GmVSP*α promoter drove the highest LUC activity, which was stronger than the constitutive CaMV35S promoter. This finding suggests that the *GmVSP*α promoter may be more constitutive than inducible, particularly due to the presence of a clear TA-rich region (which is missing in all of the others). In contrast, the *GmVSP*β promoter produced a lower LUC activity than the CaMV35S promoter, and the *GmN:IFR* promoter yielded the lowest LUC activity, suggesting that *GmVSP*β and *GmN:IFR* most likely represent inducible promoters related to the induced defense mechanism in soybean.

The inducible characteristic of the *GmVSP*β and *GmN:IFR* promoters was further supported by the results from co-transfecting each promoter (reporter) and TF (effector) in *Arabidopsis*: the LUC activity of these two promoters was strongly affected by a range of co-transfected effectors containing the 12 selected TFs driven by the CaMV35S promoter. Importantly, even TFs belonging to the same family had different effects on the activity of the *GmVSP*β and *GmN:IFR* promoters. As shown in **Figures 4B,C**, *GmVSP*β promoter activity was significantly increased (by at least two-fold) by 11 of the 12 co-transfected effectors, the exception being the *GmMYB73* effector. The discrepant effect of GmMYB73 on the regulation of the GmVSPβ promoter verified that GmMYB73, as a small size protein, may need to physically interact with other TFs rather than function independently (Liu et al., 2014b). Overall, the most predominant transcriptional activators of the *GmVSP*β promoter were *GmbZIP110* and *GmWRKY39*, which yielded relative increases in induction of 5.7 fold and 4.8 fold, respectively. The results for the *GmN:IFR* promoter were similar; all 12 TF effectors highly significantly enhanced the *GmN:IFR* promoter activity with greater strength and fold induction (3.5 fold to 8.5 fold). The co-transfection of the *GmN:IFR* promoter with the *GmWRKY39, GmWRKY20* or *GmMYB75* TF effector all yielded the highest LUC activity, with a greater than eight fold induction. Both the *GmVSP*β and *GmN:IFR* promoters showed good inducibility by a range of previously co-expressed TFs and were more specific for different predominant transcriptional activators, which is consistent with the presence of different numbers of putative TFBSs within their nucleotide sequence. In addition, considering the functional conservation of the *GmVSP*β and *GmN:IFR* genes in insect resistance in transgenic tobacco, this observation strongly suggests that the corresponding TFs may play general and crucial roles in the coordinated regulation of these genes during stress and defense-related responses. In contrast, the *GmVSP*α promoter, which was shown to contain fewer putative TFBSs, may not be recognized by these TFs. Only two of the 12 TFs significantly activated *GmVSP*α promoter activity, leading to 2.1-fold and 1.4-fold increases in the expression of the LUC reporter gene, respectively. Four TFs, including *GmMYB50, GmNAC3, GmWRKY20* and *GmWRKY28*, failed to significantly alter *GmVSP*α promoter activity. The remaining six TFs clearly reduced the LUC activity driven by the *GmVSP*α promoter (**Figure 4A**), which was quite different from the promoters of its isoforms, *GmVSP*β and *GmN:IFR*. This result is consistent with the distinct expression patterns mediated by these promoters in response to CCW feeding, as previously reported by microarray studies, as well as the predicted numbers of putative elements in those promoters.

Our work provides the first clear evidence that combinatorial interactions occur between representative functional genes and multiple relevant TFs in response to CCW feeding in soybean to regulate the induction of resistance. These results help elucidate how variation within promoters affects gene expression and, ultimately, phenotypic diversity in different lines, and they explain why the resistant line exhibited stronger and longer-lasting induced resistance than the susceptible line. Due to their combinations of different elements and their proposed interactions with different TFs, the *GmVSP*β and *GmN:IFR* promoters may be among the best insect-inducible promoters that directly regulate herbivore-induced defense responses in soybean. However, genes regulated via different TFs have also been identified in other studies. One example is the *gst1* promoter, which contains both a W box and an S box and is regulated under the control of both WRKY and AP2/ERF TFs (Strittmatter et al., 1996). The discovery of TF genes associated with the activity of the *GmVSP*β and *GmN:IFR* promoters is important because they are likely to orchestrate the genome-wide changes in transcription that lead to complete functional gene-mediated CCW resistance in soybean. These findings might, however, represent only a fraction of all the TFs involved in defense against herbivory; our model does not exclude possible contributions of additional factors.

### GmbZIP110 Specifically Activates the Transcription of *GmVSP***α***/***β**

*GmVSP*α and *GmVSP*β, two soybean VSPs with 80% amino acid homology, have been demonstrated to share similar expression patterns in response to various external stimuli, such as wounding, water deficit, sugars, JA treatment and insect herbivory (Mason and Mullet, 1990). Our previous transcriptome studies showed that *GmVSP*β was induced by insects in two soybean lines; the substantially stronger induction of *GmVSP*β in the resistant soybean line was accompanied by the simultaneous up-regulation of many TFs. In contrast, *GmVSP*α was induced only in the susceptible line, and no TFs were co-regulated (**Figure 1**). There is a strong chance that the different expression patterns of *GmVSP*α and *GmVSP*β are caused by the low level of identity (47.4%) between their promoters. Although VSP genes have been extensively functionally characterized in soybean and other plant species, revealing their involvement in defense responses (Staswick et al., 2001; Liu et al., 2005), the regulatory control underlying VSP gene-mediated defense mechanisms remains to be elucidated. This study, which compared the promoter sequences of *GmVSP*α and *GmVSP*β, revealed different copy numbers of regulatory elements between the two VSP genes, which led to different results from co-transfection assays, where *GmVSP*β promoter activity was positively regulated by 11 different TFs, and *GmVSP*α promoter activity was enhanced by only two TFs. However, it is noteworthy that of all 12 TF effectors, *GmbZIP110* showed the greatest ability to transactivate both *GmVSP*α (2.1 fold) and *GmVSP*β (5.7 fold) promoter activities (**Figures 4A,B**). This result is consistent with the finding of significant overrepresentation of the same number of bZIP-binding sites in their promoters (**Table 2**).

**FIGURE 5:**
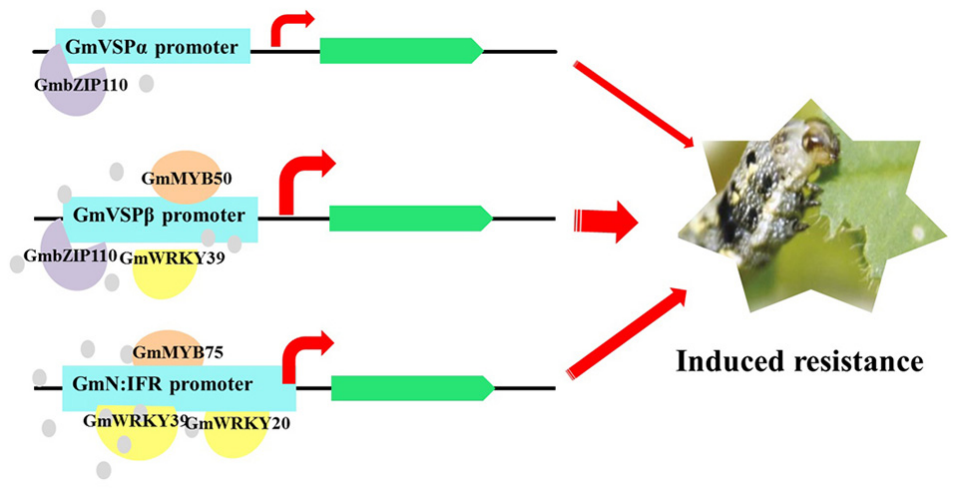
**Proposed model of regulatory transcription factors directly activates the key function genes, which lead to an effective and specific herbivore induced defense response in the resistant soybean lines as previously reported (Wang et al., 2014).** The results highlighted predominant roles of *GmbZIP110, GmMYB75*, which plays a distinct role in the activation of *GmVSP*α*/*β promoter and *GmN*:*IFR* promoter, respectively, and *GmWRKY39*, which acts as a general transcriptional activator of all the three genes tested. This model only contains TFs described in this study. The thickness of each arrow represents the extent of predicted contributions from each of the regulated functional genes.

Transcription factors play an essential role in the abiotic stress response by regulating the mRNA abundance of a large spectrum of downstream target genes via interactions with *cis*-acting elements in the promoters of these genes (Liu et al., 2014a). Some of these TFs regulate various stress-inducible genes either cooperatively or separately, while others specifically regulate only one or several homogeneous genes. Here, we identified a unique TF, *GmbZIP110*, that specifically increased the promoter activities of two soybean VSP genes. bZIP proteins, one of the most diverse families of TFs, regulate plant development, physiological processes, and biotic/abiotic stress responses (Baloglu et al., 2014). However, only a few soybean bZIP TFs have been functionally analyzed, and virtually nothing is known regarding their roles in herbivore-induced defense. *GmbZIP110* belongs to the salt-responsive group S bZIP1 superfamily and is similar to the well-characterized maize *OCSBF-1* and *Opaque2* genes and the *Arabidopsis AtZIP1* gene. The *Arabidopsis AtbZIP1* TF is a positive regulator of plant tolerance to salt, osmotic and drought stresses. Similar to *GmVSPs*, the expression of *bZIP1* is regulated by sugars (Kang et al., 2010; Dietrich et al., 2011) and upregulated at both the transcriptional and posttranscriptional levels by energy depreciation (Dietrich et al., 2011). In addition, *Opaque2* is a known transcriptional activator of the maize zein gene, which is also reported to encode the prolamin seed storage protein (Vicente-Carbajosa et al., 1997). Based on our study and the above information, it is tempting to speculate that the *GmbZIP110*-mediated regulation of *GmVSP*α*/*β is a part of a specific and critical regulatory mechanism that integrates induced defense responses and energy availability through sugar signaling (**Figure 5**).

### *GmMYB75* Specifically Activates the Transcription of *GmN:IFR*

MYB TFs constitute a diverse class of DNA-binding proteins with particularly important roles in transcriptional regulation in plants. MYB TFs are characterized by the presence of 1–3 imperfect repeats (R1, R2, and R3) of the MYB domain (Jin and Martin, 1999). Single MYB-domain proteins bind DNA differently compared with two-repeat or three-repeat MYB proteins; thus, they have different functions (Jin and Martin, 1999). The roles of individual MYB proteins have been extensively investigated in diverse plants (Quattrocchio et al., 1998). A significant number of these MYB TFs are involved in the precise regulation of secondary metabolism, particularly phenylpropanoid metabolism, including branches of anthocyanin production (Schwinn et al., 2006), flavonol biosynthesis (Mehrtens et al., 2005), lignin biosynthesis (Patzlaff et al., 2003) and isoflavonoid biosynthesis (Yi et al., 2010), which provides various secondary metabolites related to abiotic and biotic stresses (Vogt, 2010). The different branches of phenylpropanoid metabolism are differentially controlled by distinct MYB factors, and these factors function via different modes of action. However, in legumes, the extra dimension of the regulatory control of the most-downstream branch of phytoalexin biosynthesis is not fully understood. *GmN:IFR* encodes an NADPH: isoflavone reductase, the key enzyme involved in phytoalexin biosynthesis (Tiemann et al., 1991). A transcriptome analysis revealed that the expression of *GmN:IFR* was induced in response to insect feeding, along with a group of TFs that included nine MYBs. A promoter analysis revealed that the *GmN:IFR* promoter contains at least twice as many MYB-binding sites as both *GmVSP*α*/*β promoters (**Table 2**). Consistent with this finding, the co-transfection analysis demonstrated that all three tested *GmMYBs* significantly induced the *GmN:IFR* reporter activity at levels 3.6- to 8.3-fold greater than the control (**Figures 4B,C**). Among these *GmMYBs, GmMYB50*, and *GmMYB75* also significantly induced the *GmVSP*β reporter activity, albeit with lower FCs. *GmMYB75* produced the most marked effect on the *GmN:IFR* promoter (8.3 fold), which was prominently disparate from its effect on the *GmVSP*β promoter (2.3 fold). Similar to most plant MYBs, *GmMYB50* and *GmMYB75* contain two repeats (R2R3 MYB proteins), whereas the less effective gene, *GmMYB73*, only contains one MYB domain. This observation suggests that single MYB-domain proteins bind DNA in a different way than two-repeat MYB proteins and are likely to have different functions, as reported elsewhere (Jin and Martin, 1999). The least-related TF, *GmMYB73*, has been found to function in lipid accumulation (Liu et al., 2014b). A phylogenetic analysis of *GmMYB50* and *GmMYB75* demonstrated that *GmMYB50* was similar to *AtMYB44* (**Figure 2**), which has been reported to play a role in abiotic stress tolerance (Jung et al., 2008), and *GmMYB75* was close to a development-related gene in *Antirrhinum, AmDIV* (Galego and Almeida, 2002). Nevertheless, our results imply that the *GmMYB50* protein plays a general regulatory role by transactivating two defense genes, and they highlight a specific regulatory role of *GmMYB75* in transactivating *GmN:IFR*. Thus, our data support important and distinct roles of these two MYB factors in mediating CCW resistance, primarily through the regulation of phytoalexin biosynthesis (**Figure 5**).

### GmWRKY39 Generally Activates the Transcription of GmVSPs and GmN:IFR

Recently, transcriptome research on soybean defense responses has demonstrated the convergence of functional genes and regulatory genes at the level of transcriptional activation. Transcriptome analyses have revealed a total of at least 45 TFs that are activated during the incompatible soybean-CCW interaction, including WRKYs, NACs, MYBs, bZIPs and members of several other TF families. The 17 members of WRKY TFs constitute the single largest class in this group, indicating that WRKY factors may play a major role in transcriptional reprogramming during the defense response to CCW. WRKY proteins can be classified into three groups (I, II, and III) based on the number of WRKY domains at the N-terminus and the pattern of the zinc-finger motif at the C-terminus (Eulgem et al., 2000). Four WRKY TFs that are not closely related, including *GmWRKY28, GmWRKY21, GmWRKY20*, and *GmWRKY39*, were selected for co-transactivation assays with three functional gene promoters (**Figure 4**). Based on sequence similarity, *GmWRKY28* and *GmWRKY21* are both classified as WRKY group IIc, similar to three soybean WRKY group IIc genes that were found to be induced by soybean aphid in a resistant cultivar, but not in a susceptible cultivar (Li et al., 2008). *GmWRKY20* is a member of WRKY group III, as is rice *WRKY89*, which has been implicated in resistance to the phloem-feeding whitebacked leafhopper (Wang et al., 2007). These two types of WRKYs are largely associated with sucking insect defense, which resembles a defense against pathogens more closely than a defense against chewing insects. However, the last gene, *GmWRKY39* is a member of WRKY group I, as are tobacco *WRKY3* and *WRKY6*, which play important roles in resistance to the chewing insect Manduca sexta via the JA signaling pathway in tobacco plants (Skibbe et al., 2008). Although both *GmWRKY21* and *GmWRKY20* have been implicated in abiotic stresses, only *GmWRKY39* has been reported to show an induced response to *Sclerotinia sclerotiorum* inoculation. This striking connection between *GmWRKY39* and biotic stresses, particularly chewing insect defense, is highly consistent with our co-transactivation assay results. In that assay, *GmWRKY39* significantly activated the *GmVSP*α promoter (1.4 fold) and highly significantly activated the *GmVSP*β (4.8 fold) and *GmN*:*IFR* promoters (8.5 fold); all three FCs were the second largest among the 12 TFs. The other three GmWRKYs, which are less closely related, only yielded modest activation of two of the promoters (**Figure 4**). These results are consistent with the discovery of greater numbers of putative binding sites for WRKY factors (W box) in the *GmVSP*β and *GmN*:*IFR* promoters compared with *GmVSP*α (**Table 2**). Although the specific transactivation effect of each TF varied depending on its interactions with the promoters of different genes, *GmWRKY39* was the only TF that significantly highly activated all three promoters, suggesting that *GmWRKY39* alone can modulate the expression levels of genes involved in multiple mechanisms and thus plays a prominent role in the soybean defense response to chewing insects, particularly CCW (**Figure 5**).

## Conclusion

The combinatorial control of defense genes via interactions with multiple TFs is one of the most important characteristics of transcriptional regulation in plant defense systems (Rushton and Somssich, 1998). Our study revealed general and distinct interactions between three previously isolated soybean defense-related genes and selected co-expressed TFs, thus providing a comprehensive understanding of the complex regulatory networks and defense gene activation involved in soybean induced defense responses, which may be the main source of the effective and specific herbivore-induced defense response previously reported in resistant soybean lines. Two of the three gene promoters, *GmVSP*β and *GmN*:*IFR*, were demonstrated to be strong inducible promoters containing numerous putative defense/stress-related TFBSs. The activities of these two promoters were both significantly enhanced by nearly all of the tested TFs when they were co-transfected into *Arabidopsis thaliana* protoplasts. The high induced efficiency of the *GmVSP*β and *GmN*:*IFR* promoters in regulating gene expression is important for engineering plants with increased insect resistance by facilitating the highly restricted expression of gene products only upon insect attack. Detailed analyses of these two promoters should be conducted to identify the precise regulatory *cis*-elements that are responsible for the interactions between the promoters and specific TFs. In addition, this study compared the involvement of 12 TF genes that are likely involved in CCW resistance in the resistant soybean line. The results highlighted the predominant roles of *GmbZIP110* and *GmMYB75*, which play distinct roles in the activation of the *GmVSPα*/*β* promoter and the *GmN*:*IFR* promoter, respectively, and *GmWRKY39*, which acts as a general transcriptional activator of all three tested genes. Together, these data help explain the divergence and conservation of defense-responsive regulatory networks and suggest that these specific TFs may act early in the related defense gene-mediated CCW resistance pathway (**Figure 5**), which makes them important targets for further functional analyses to fully understand the roles of these TF networks. Overall, the work presented here constitutes a critical preliminary step toward identifying regulatory nodes in the transcriptional network of insect-induced resistance in soybean, and the results represent a framework for future studies aimed at reconstructing higher-order gene regulatory networks in plants.

## Author Contributions

YW designed and performed research, analyzed data and wrote the manuscript. HW and QY participated in the improvement of the manuscript. YM participated in the gene cloning and plasmid construction of the promoters and TF genes. HD participated in co-transactivation experiment. DY designed the research and revised the manuscript. All the authors have read and approved the final manuscript.

## Conflict of Interest Statement

The authors declare that the research was conducted in the absence of any commercial or financial relationships that could be construed as a potential conflict of interest.

## Footnotes

1 http://www.ncbi.nlm.nih.gov/geo/

2 http://www.phytozome.net/soybean/

3 http://www.ncbi.nlm.nih.gov/cdd

4 http://psort.nibb.ac.jp/

5 http://www.cbs.dtu.dk/services/ProtFun/
