# Supplementary material for: Identification of transcriptional regulatory nodes in soybean defense networks using transient co-transactivation assays
Source: Front Plant Sci. 2015 Oct 27;6:915. doi: 10.3389/fpls.2015.00915 (PMC4621403; doi:10.3389/fpls.2015.00915)
Supplement: Supplementary file 1 [file Data_Sheet_1.DOC]

Identification of transcriptional regulatory nodes in soybean defense networks using transient co-transactivation assays. Frontiers in Plant Science. Yongli Wang, Hui Wang, Yujie Ma, Haiping Du, Qing Yang, and Deyue Yu*

*Corresponding author.

E-mail address: dyyu@njau.edu.cn

College of Agriculture, Nanjing Agricultural University, Nanjing 210095, China

Tel.: +86-25-84396410

Fax: +86-25-4395110

Figure S1

Fig. S1 Map of the constructed pCaMV35S-*LUC* plasmid

Figure S2


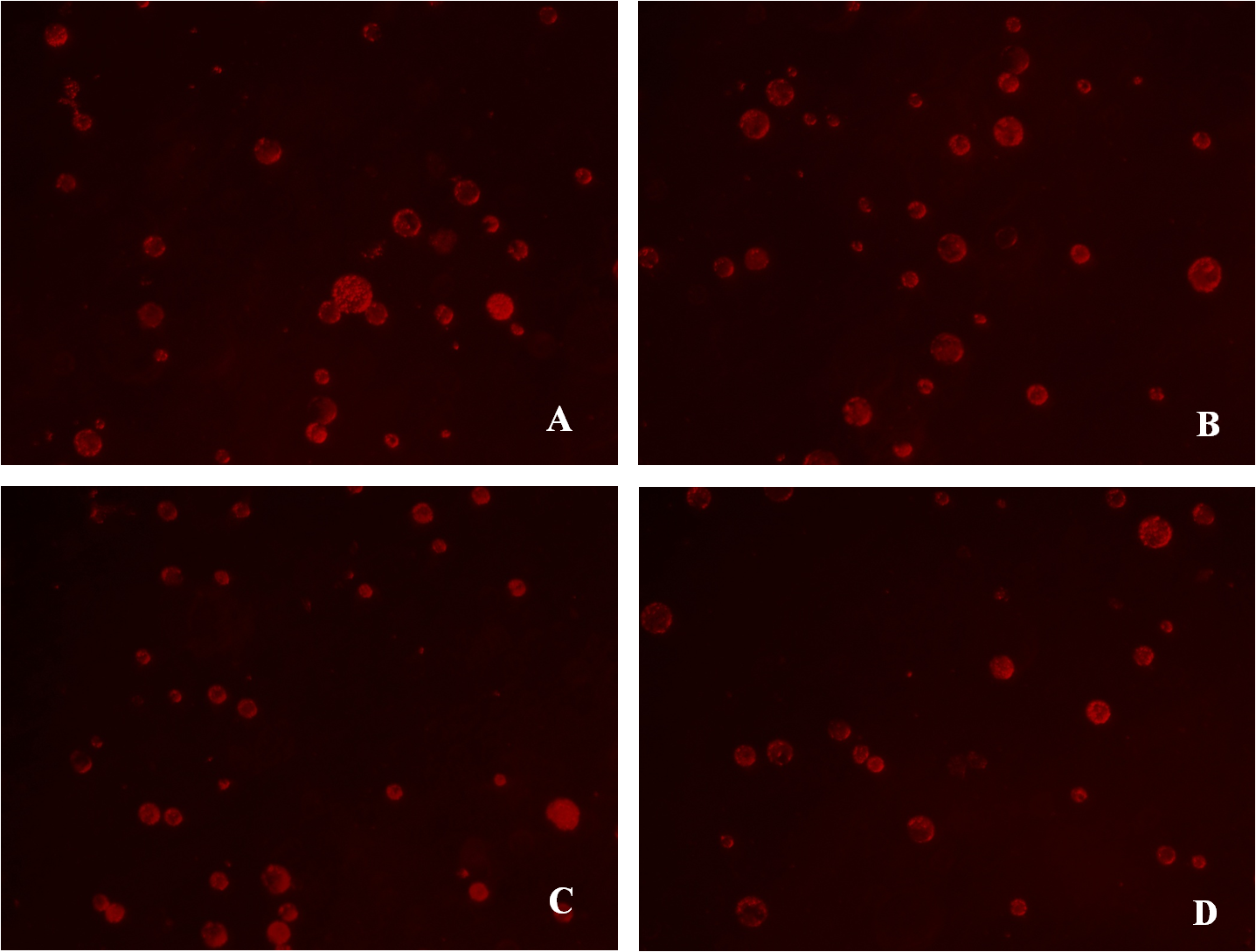


Fig. S2 Four field views of protoplasts isolated from Arabidopsis thaliana under fluorescence microscopy (×400)

**Table S1.** Primers used for cDNA cloning and plasmid construction of the 12 transcription factors

| **Gene title** | | **Forward primer (5′→3′)** | **Reverse primer (5′→3′)** | **Length** |
| --- | --- | --- | --- | --- |
| *GmWRKY39*  *GmWRKY21*  *GmWRKY28* | Sequence clone | GAGACAACGCTTTCAAAACGC | TTCGCTGCTCCACTTCCACAT | 918bp |
|  | plasmid construct | GAGAGAACACGGGGGACTCCATGGAAGACAAAGACAGAGC | TCGCTGAATACAGTTACATTTCATCTGATGCGCATTCCCG | 937bp |
|  | Sequence clone | GCTATTACCTCAATGGATTACTAT | ATATTACTGTATTGAAGAGGATCA | 624bp |
|  | plasmid construct | GAGAGAACACGGGGGACTCCATGGATTACTATTTTGGAAA | TCGCTGAATACAGTTACATTTCATGAGTTTGCAGAAGGGT | 631bp |
|  | Sequence clone | ATGGCATCTTCTTCTGGTAGTTTA | TCCTTCAGCATAGTAGAGACTCAA | 1741bp |
|  | plasmid construct | GAGAGAACACGGGGGACTCCATGGCATCTTCTTCTGGTAG | TCGCTGAATACAGTTACATTTCAGCATAGTAGAGACTCAA | 1783bp |
| *GmWRKY20* | Sequence clone | AGAAGAGCATCATCTACGACCATT | GTCCCGTTTTATGAAGACTACATT | 1181bp |
|  | plasmid construct | GAGAGAACACGGGGGACTCCATGGAGAGTGACTTGAGCTG | TCGCTGAATACAGTTACATTTCAGCACAAAAATCCTGGGG | 1120bp |
| *GmbZIP110* | Sequence clone | CATTGCACAAATTAAATAGC | CAAAAACTCCAACTCAAAAT | 569bp |
|  | plasmid construct | GAGAGAACACGGGGGACTCCATGGCTTCTCCTGGTGGAAG | TCGCTGAATACAGTTACATTTCAATACATCATCAACATGT | 547bp |
| *GmDREB1* | Sequence clone | GCAAAGAAAGATAAGATAGGT | GATTGAAGATACGGAAAAACA | 824bp |
|  | plasmid construct | GAGAGAACACGGGGGACTCCATGGAAGACAGGGATCACTG | TCGCTGAATACAGTTACATTTCAATCTTGAAGCTCTTCGA | 565bp |
| *GmNAC34* | Sequence clone | AATGAGCAACATAAGCATGGTAGA | GTCTCACATCAGTAATTCCACACG | 918bp |
|  | plasmid construct | GAGAGAACACGGGGGACTCCATGAGCAACATAAGCATGGT | TCGCTGAATACAGTTACATTTCAGTAATTCCACACGTGGG | 949bp |
| *GmNAC3* | Sequence clone | ATGGGAGTTCCAGAGAGAGA | TCACTTCACTCCCTAACCCC | 1007bp |
|  | plasmid construct | GAGAGAACACGGGGGACTCCATGGGAGTTCCAGAGAGAGA | TCGCTGAATACAGTTACATTTCACTCACTCCTACCCGACC | 1047bp |
| *GmNAC26* | Sequence clone | CCTCTCTTGTCCCAATAATTAGCA | TTTTTTTGGCATTATCCTCTTTGA | 945bp |
|  | plasmid construct | GAGAGAACACGGGGGACTCCATGGAGAACAGAACAAGCTC | TCGCTGAATACAGTTACATTTTATCCTCTTTGATAATACA | 880bp |
| *GmMYB50* | Sequence clone | TTCAATTTCCTCTCACAGGCTTTA | TTTCTCCGATGATACTATTTACGA | 959bp |
|  | plasmid construct | GAGAGAACACGGGGGACTCCATGGACCGGATCAAAGGCCC | TCGCTGAATACAGTTACATTTTACGAATCAACCCTGCTAA | 934bp |
| *GmMYB73* | Sequence clone | ATGGCTGACATAGATCGCTC | CTGTAATGACTTGTAGGCAC | 267bp |
|  | plasmid construct | GAGAGAACACGGGGGACTCCATGGCTGACATAGATCGCTC | TCGCTGAATACAGTTACATTTCATTGGCTAGTCGAAAATC | 265bp |
| *GmMYB75* | Sequence clone | TTTTTGTATGCTTGTGGCTTGAGA | CATCATCAGGGTTGGCATTACAGT | 963bp |
|  | plasmid construct | GAGAGAACACGGGGGACTCCATGGAATTGGAAACCATCTA | TCGCTGAATACAGTTACATTTTACAGTGCATGAATACCAA | 961bp |

**Table S2.** Primers used for cloning and plasmid construction of *GmVSPα*, *GmVSPβ* and *GmN:IFR* gene promoters

| **Gene title** | | **Forward primer (5′→3′)** | **Reverse primer (5′→3′)** | **Length** |
| --- | --- | --- | --- | --- |
| *GmVSPα* promoter  *GmVSPβ*promoter  *GmN:IFR* promoter | Sequence clone | TTCAGATTTAAGTGATAAGAGTGGAC | CGAGGTCTCTGGCATAAAAGTAA | 1450bp |
|  | plasmid construct | CCTGCAGGCTCTAGAGGATCCTTCAGATTTAAGTGATAAGA | TGTTTTTGGCGTCTTCCATGGCACACTGTTTCTAGTTATCT | 1206bp |
|  | Sequence clone | TAACTTGCTAACTCGTTGACTCGGTG | AAATATGTCGTTGTGATGGACTTCG | 1981bp |
|  | plasmid construct | CCTGCAGGCTCTAGAGGATCCTAACTTGCTAACTCGTTGAC | TGTTTTTGGCGTCTTCCATGGATTCTCCTCACGATAAACTA | 1711bp |
|  | Sequence clone | ATGGTACGGGTGTTGTGATAATTAAT | AAACTTGCCCAAACTATGTGCCTTC | 1812bp |
|  | plasmid construct | CCTGCAGGCTCTAGAGGATCCATGGTACGGGTGTTGTGATA | TGTTTTTGGCGTCTTCCATGGGGAAGATACTAGTCTAAACA | 1780bp |

**Table S3.** Accession numbers of homologous transcription factors used in the phylogenetic tree construction in Figure 2.

| Gene Name | Accession number | Gene Name | Accession number | Gene Name | Accession number |
| --- | --- | --- | --- | --- | --- |
| *AtWRKY57* | U92266 | *AtWRKY51* | U92267 | *AtWRKY33* | AF006195 |
| *AtWRKY53* | U50768 | *AtZIP1* | AF139205 | *AtRAP2-1* | U87908 |
| *AtNAC22* | AF139206 | *AtNAC72* | AAS47693 | *AtNAC19* | AAD19839 |
| *AtCPC* | FJ797957 | *AtNAC29* | AAC39443 | *AtMYB44* | AAD34294 |
| *CaNAC72* | XP_004514350.1 | *GaNAC72* | KHG15675.1 | *CaNAC29* | XP_012572610.1 |
| *CaWRKY43* | XP_004502873.1 | *CaWRKY57* | XP_004490148.1 | *CaWRKY41* | XP_012572172.1 |
| *GsNAC29* | KHN14494.1 | *GsRAP2-1* | KHN12801.1 | *GsCPC* | KHN41203.1 |
| *GsMYB1R1* | KHN20871.1 | *GsWRKY51* | KHN09555.1 | *AhMYB25* | AHB59611.1 |
| *AhMYB20* | AHB59606.1 | *AhMYB29* | AHB59615.1 | *MtMYB51* | XP_003616568.1 |
| *AmDIV* | L37750 | *AmDREB* | AHI96435.1 | *MtbZIP124* | XP_003629740.2 |
| *OCSBF-1* | AF139207 | *GhWRKY41* | ADZ99351.1 | *GhWRKY33* | AIE43867.1 |
| *GhWRKY67* | AIE43901.1 | *GmDREB2* | CDU32445.1 | *PvNAC72* | AGV54694.1 |
| *MeNAC32* | ALC79009.1 | *PtNAC1* | XP_002310688.1 | *RcNAC21* | XP_002529954.1 |
| *HhDREB* | ACJ66376.1 | *TcbZIP44* | XP_007042116.1 | *GaWRKY33* | KHG00824.1 |
